# Hypoxia Increases Cardiac Proteasomal Activity and Differentially Modulates Cullin-RING E3 Ligases in the Naked Mole-Rat *Heterocephalus glaber*

**DOI:** 10.3390/muscles5010006

**Published:** 2026-01-14

**Authors:** W. Aline Ingelson-Filpula, Karen L. Kadamani, Mohammad Ojaghi, Matthew E. Pamenter, Kenneth B. Storey

**Affiliations:** 1 Clark H Smith Brain Tumour Centre, University of Calgary, Calgary, AB T2N 1N4, Canada; 2 Arnie Charbonneau Cancer Institute, University of Calgary, Calgary, AB T2N 1N4, Canada; 3 Alberta Children’s Hospital Research Institute, University of Calgary, Calgary, AB T2N 1N4, Canada; 4 Department of Biochemistry and Molecular Biology, University of Calgary, Calgary, AB T2N 1N4, Canada; 5 Biology Department, University of Ottawa, Ottawa, ON K1N 6N5, Canada; kkada037@uottawa.ca (K.L.K.); mojag071@uottawa.ca (M.O.); mpamente@uottawa.ca (M.E.P.); 6 Ottawa Institute of Systems Biology, University of Ottawa, Ottawa, ON K1N 6N5, Canada; 7 Department of Biology, Carleton University, Ottawa, ON K1S 5B6, Canada

**Keywords:** ubiquitin-mediated proteolysis, degradation, muscle, extreme environmental stress

## Abstract

(1) Background: The naked mole-rat (*Heterocephalus glaber*) survives hypoxia–reoxygenation stresses by utilizing metabolic rate depression, achieved in part by downregulating nonessential genes and processes to conserve endogenous cellular resources and prevent buildup of toxic waste byproducts. Tight molecular control of protein degradation (specifically the ubiquitin–proteasome system) is a potent regulatory tool for maintaining muscle integrity during hypoxia, but how this system is regulated in the heart of hypoxia-tolerant species is poorly understood. (2) Methods: The protein expression levels of cullin-RING E3 ligases (specifically CRL4 architecture), deubiquitinating enzymes, and proteasomal activity were assayed in cardiac tissues from *H. glaber* exposed to 24 h of normoxia or hypoxia in vivo. (3) Results: Overall, the protein expression of E3 ligases decreased, whereas expression of deubiquitinating enzymes increased during hypoxia, all of which play roles in themes of oxidative stress, heightened DNA damage repair, and the HIF-1-VHL-NFκB axis. Proteasomal activity was elevated during hypoxia, which conceivably links to the oxidative stress theory of aging and longevity of *H. glaber*. (4) Conclusions: Taken together, our results expand current research into protein degradation and extreme environmental stress responses, with a specific focus on cardiac mechanisms related to oxidative stress resistance along the hypoxia-longevity axis.

## 1. Introduction

Hypoxia exposure, or a condition of suboptimal oxygen availability, is linked to a myriad of deleterious physiological changes, including impaired aerobic metabolism and oxidative stress generation [1,2,3,4,5]. Oxidative stress is primarily caused by reactive oxygen species (ROS) production-free radicals of oxygen that can lead to fatty acid peroxidation and DNA/RNA damage in the form of double bond adducts [6,7,8]. Tissues with high metabolic demand are at particular risk for hypoxic damage, exemplified clinically by the severity of myocardial infarction/cardiac arrest [9], hypoxia during fetal development linked with adverse cardiac development [10,11], and studies that show hypoxia-induced inflammation and injury in cardiac myocytes [12]. Even anaerobic-permissive tissues such as skeletal muscle are susceptible to damage, as exemplified by muscle atrophy/wasting in both chronic respiratory disease [13,14] and chronic obstructive pulmonary disease [15,16], as well as myofibrillar filament loss from impaired muscle regeneration post-hypoxic injury [17,18]. Molecular pathways implicated in the aforementioned conditions share many commonalities: metabolic underpinnings, including glycolytic shifts coupled with impaired oxidative metabolism/phosphorylation; mammalian/mechanistic target of rapamycin complex 1 (mTORC1) dysregulation; AMP-activated protein kinase (AMPK) activation; and upregulation of ubiquitin-mediated proteolysis [17,19].

Altered metabolism and the balance between protein synthesis and protein degradation are hallmarks in animals that mitigate hypoxia exposure as part of their lifestyle, which often and remarkably demonstrates low to no substantial muscle atrophy. One such species is the naked mole-rat (*Heterocephalus glaber*), presumed to regularly encounter intermittent hypoxia in poorly ventilated subterranean burrows, which constitute its natural habitat, similar to other mole-rat species [20,21,22]. The true extent of oxygen fluctuation has not been rigorously identified due to the irregular nature of these hypoxic sections of burrow; nevertheless, it is indisputable that hypoxia tolerance is a crucial adaptation for *H. glaber* given its robust ability to survive chronic hypoxia/acute anoxia in laboratory settings and the identification of many physiological/molecular adaptations that facilitate hypoxia endurance [23,24,25,26,27,28]. Thus, naked mole-rats are one of many animal species in nature that contend with extreme environmental stresses as part of their lifestyle [29], which have evolved extensive (but reversible) changes in their phenotype to survive these conditions. These changes, i.e., a suite of molecular processes, are collectively grouped together as metabolic rate depression (MRD) and facilitate a global downregulation of nonessential genes and proteins to conserve cellular energy, aided by other changes, including altered fuel metabolism, modulations in enzyme activity, increased antioxidant defenses, and limitation of waste buildup from metabolic end products [29,30]. *H. glaber* has a naturally low resting metabolic rate but likewise employs MRD to deal with its frequent exposure to hypoxia in its burrows [31]. Under hypoxia, naked mole-rats depress their metabolic rate by up to 85%, reduce heart and respiratory rate, and switch to anaerobic modes of metabolism, to name a few [26,31,32,33]. They possess cardiometabolic adaptations for hypoxia, such as elevated glycogen and reduced succinate to resist ischemic damage [34], as well as lactate-mediated resistance to damage caused by impaired mitochondrial respiration [35,36]. Naked mole-rats are one of the few species to maintain a level of activity during MRD/hypoxic exposure despite their lowered T_b_, heart rate, and metabolic rate, whereas other species are completely immobile during hypometabolic periods [31,37]. Mechanisms that preserve mole-rat skeletal muscle integrity during hypoxia are often shared in cardiac muscle as well [38,39].

Animal species with MRD-facilitated adaptations for surviving hypoxia are intriguing avenues for research from a human clinical perspective, with several introductory studies proposing controlled hypoxia for pre-conditioning or cardioprotective strategies [40,41,42,43]. While *H. glaber* has been studied primarily as a longevity model [44,45,46,47], there has been introductory research into skeletal muscle protein synthesis specifically during hypoxia [48]. Investigation of specific mechanisms of protein degradation, however, is far less comprehensive despite the ubiquitin–proteasome system (UPS) being responsible for 80–90% of cytosolic protein degradation [49]. Protein degradation (and control thereof) by tagging substrate proteins with ubiquitin to direct them for breakdown within the proteasome is deeply implicated in maintaining cellular homeostasis by regulating the cell cycle, signaling networks, and DNA damage repair, as well as interfacing with other forms of epigenetic regulation, including modifying histone proteins, tagging with small ubiquitin-like modifier (SUMOlyation), and tagging with ubiquitin-like NEDD8 (NEDDylation) [50,51]. The recyclability of ubiquitin to maintain a stable cellular pool, along with its ability to form variable mono- and polyubiquitin chains on target lysines, allows ubiquitination to be a rapid and versatile regulatory method [52,53,54,55,56].

Ubiquitin, a small 72 amino acid protein, is converted to its functional form by ubiquitin-activating E1 enzymes before forming a complex with ubiquitin-conjugating E2 enzymes to catalyze the binding of ubiquitin to substrate protein via target-specific E3 ligases [57]. There are over 600 human E3 ligases grouped into the Homologous to the E6-AP Carboxyl Terminus (HECT), Really Interesting New Gene (RING) finger, and Ring-Between-Ring (RBR) families [51], with over 200 members in the cullin-RING E3 ligase (CRL) superfamily responsible for ~20% of all ubiquitination in cells [58,59]. The architecture of CRLs consists of the following four components: a cullin protein that acts as a scaffold; a RING finger protein (either RING-box protein 1 or 2 [RBX1/2]) that binds to an E2 ubiquitin-conjugating enzyme; an adaptor protein that bridges substrate receptor to cullin; and a substrate receptor to recognize the target protein (and are in turn specialized for various adaptor proteins) [60]. Mammals express seven canonical cullin proteins [61], with various cullin-adaptor protein linkages associated with various cellular outcomes; CRL1^β-TrCP1^ is associated with cell adhesion and signaling, CRL1^Skp2^ and CRL4^Cdt2^ are implicated in cell division, CRL2^VHL^ is linked with hypoxic response, CRL3^Keap1^ is coupled with oxidative stress, and CRL4^DDB-2^ is overwhelmingly involved with DNA damage response [60]. CRL2s with the substrate receptor von Hippel-Lindau protein (VHL; CRL2^VHL^) possess the notable substrates hypoxia-inducible factor 1 alpha (HIF-1α) and HIF-2α, the hallmark indicators of hypoxia/anoxia stress [62]. Indeed, VHL is the primary regulator of polyubiquitination and degradation of HIF-1α in an oxygenated cellular environment [63,64], with hypoxia stabilizing HIF-1α subunits and allowing transcription of hypoxia-sensitive genes [65,66].

There is strong reason to believe that ubiquitination is a potent regulatory tool during hypoxia. The multicatalytic proteasome has been purified and kinetically assayed in the anoxia-tolerant red-eared slider turtle *Trachemys scripta elegans*, and while there was no change in liver proteasome activity during anoxia, aerobic recovery induced an increase in caspase activity [67]. Juvenile naked mole-rats have less protein ubiquitination and higher proteasome activity compared with standard rodents [44], and adult mole-rat liver displays higher chymotrypsin-like and trypsin-like activities along with increased 20S/26S/19S proteasomal subunits compared to those of mice [45]. The links between the UPS, hypoxia, muscle maintenance, and longevity in *H. glaber* are intriguing; therefore, this study investigated the role of cullin-E3 ligases in normoxic vs. 24 h hypoxic cardiac tissue of *H. glaber*. Western immunoblotting was used to quantify the protein expression of select E3 ligases and deubiquitinating enzymes, and a luminescent assay was used to quantify proteasomal activity with three substrates (caspase-like, chymotrypsin-like, and trypsin-like). Taken together, this expands the current research into the UPS and extreme environmental stress responses, with a specific focus on cardiac mechanisms during hypoxia.

## 2. Results

### 2.1. Protein Expression Levels of Cullin-RING E3 Ligases Declined During Hypoxia

**Figure 1:**
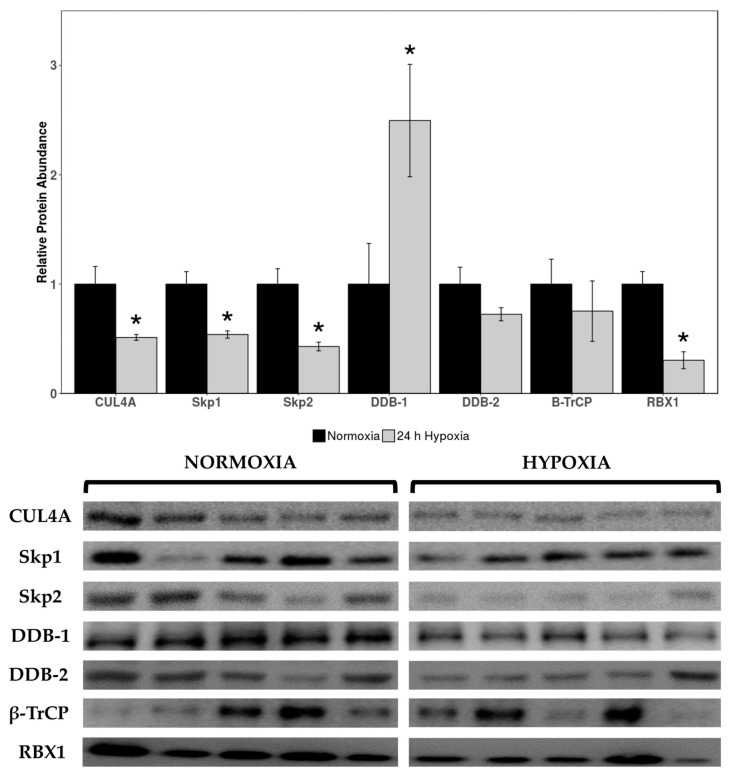
**Relative protein expression levels of cullin-RING E3 ligases in heart tissue of normoxic vs. 24 h hypoxic *H. glaber*.** Relative protein abundance of CUL4A, Skp1, Skp2, DDB-1, DDB-2, β-TrCP, and RBX1 was obtained via Western immunoblotting and is reported as mean band densities (±SEM, *n* = 4–5). Statistically significant values as determined by a Student’s *t*-test (*p* < 0.05) are denoted by an asterisk.

Relative expression levels of cardiac cullin-RING E3 ligases cullin 4A (CUL4A), seventeen kilodalton protein 1 (Skp1), Skp2, DNA damage binding 1 (DDB-1), DDB-2, beta-transducin repeat containing E3 ubiquitin protein ligase (β-TrCP), and RBX1 were quantified in normoxic vs. 24 h hypoxic *H. glaber*. There was an overall reduction in protein expression across cullin, adaptor, and RING finger proteins alike, with CUL4A decreasing to 51% of control levels during hypoxia (Figure 1) and Skp1/Skp2/RBX1 displaying an even sharper decline to 37%, 33%, and 19% of normoxic levels, respectively (Figure 1). Interestingly, there was a 2.5-fold hypoxia-induced upregulation in DDB-1 (Figure 1).

### 2.2. Upregulation of DUBs in Hypoxia-Exposed Cardiac Tissue

**Figure 2:**
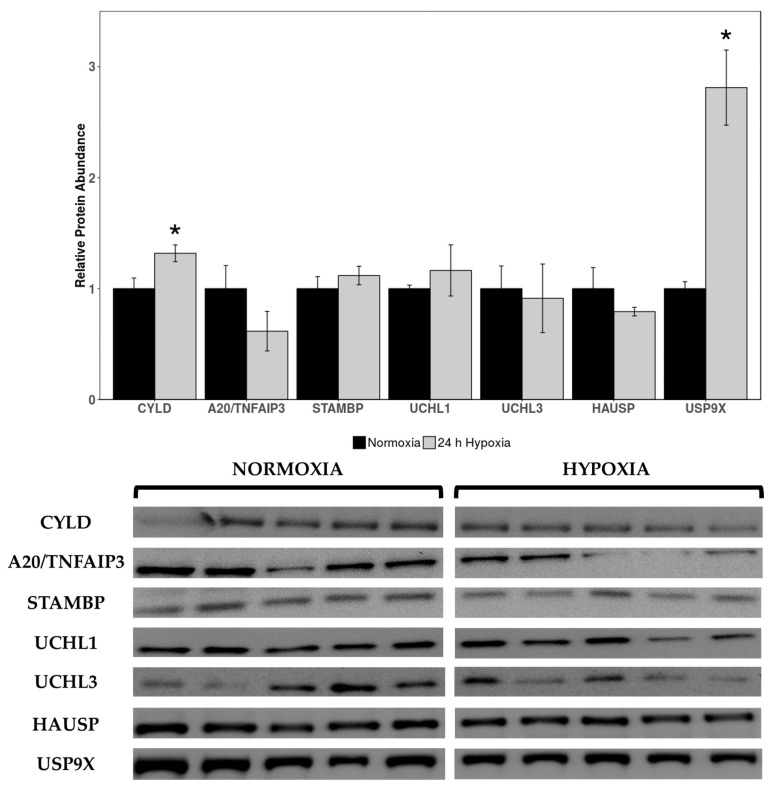
**Relative protein expression levels of deubiquitinating enzymes (DUBs) in heart tissue of normoxic vs. 24 h hypoxic *H. glaber***. Relative protein abundance of CYLD, A20/TNFAIP3, STAMBP, UCHL1, UCHL3, HAUSP, and USP9X was obtained via Western immunoblotting and is reported as mean band densities (±SEM, *n* = 4–5). Statistically significant values as determined by a Student’s *t*-test (*p* < 0.05) are denoted by an asterisk.

Western immunoblotting was used to measure levels of deubiquitinating enzymes cylindromatosis lysine 63 deubiquitinase (CYLD), pCYLD(S418), STAM binding protein gene (STAMBP), A20/TNF alpha induced protein 3 (A20/TNFAIP3), ubiquitin C-terminal hydrolase 1 (UCHL1), UCHL3, herpesvirus-associated ubiquitin-specific protease (HAUSP), ubiquitin-specific protease 10 (USP10), and USP9X in cardiac tissue of normoxic vs. 24 h hypoxic *H. glaber*. Expression of CYLD and USP9X was upregulated during hypoxia, with CYLD increasing by 1.32-fold and USP9X rising by 2.42-fold compared to normoxia (Figure 2).

No statistically significant changes were observed in other DUB protein targets between normoxia and hypoxia. Antibodies for pCYLD(S418) and USP10 did not cross-react in this species/tissue, and as such, could not be analyzed.

### 2.3. Hypoxia-Induced Upregulation in Cardiac Proteasomal Activity

**Figure 3:**
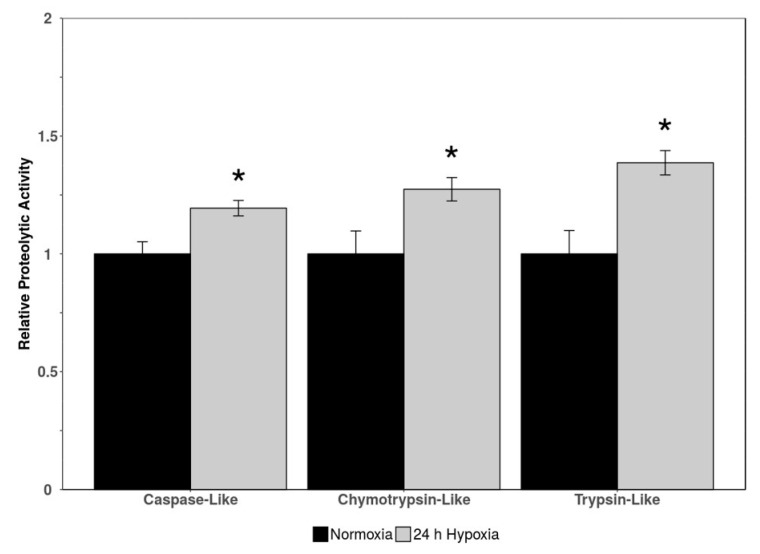
**Relative proteolytic activity (caspase-like, chymotrypsin-like, and trypsin-like) in heart tissue of normoxic vs. 24 h hypoxic *H. glaber***. Activity was determined via luminescent signal from a commercially available kit. Data is represented as mean band densities (±SEM, *n* = 4), with statistically significant values as determined by a Student’s *t*-test (*p* < 0.05) denoted by an asterisk.

Three indicators of proteasomal activity (caspase-like activity, chymotrypsin-like activity, and trypsin-like activity) were measured via luminescence in cardiac tissue of normoxic vs. 24 h hypoxic *H. glaber*. There was an upregulation in all three markers of proteolytic activity during hypoxia to 1.19-, 1.27-, and 1.39-fold for caspase-like, chymotrypsin-like, and trypsin-like activity, respectively (Figure 3).

## 3. Discussion

The UPS revolves around tagging substrate proteins with ubiquitin post-translational modifications–K48-linked chains directing proteins for degradation by the 26S proteasome and K63-linked chains serving regulatory functions in maintaining cellular homeostasis [55]. Ubiquitination has crosstalk with other forms of epigenetic modification, including histone proteins, and the UPS is responsible for 80–90% of cytosolic protein degradation [49]. Likewise, protein degradation/proteasomal activity is tightly controlled during hypometabolic states and has implications for oxidative stress responses, which may impact longevity as well as hypoxia tolerance [27,68,69]. This study examined a particular subset of RING-family E3 ubiquitin ligases, deubiquitinating (DUB) enzymes, and proteasomal activity in cardiac tissue of normoxic vs. 24 h hypoxic *H. glaber.*

### 3.1. CUL4A, Adaptor Proteins Skp1 and Skp2, and RING Protein RBX1 Were Downregulated During Hypoxia

Total protein levels of CUL4A, Skp1, Skp2, and RBX1 were downregulated during hypoxia to 51%, 37%, 33%, and 19% of normoxic levels, respectively (Figure 1). While this indicates a marginal contribution of CRL4 complexes to the hypoxic response, there are innate links with hypoxia/HIF-1α and CRL2/CRL3 ligases specifically [62,70]. VHL box proteins are the substrate receptors for elongin BC (EloBC) adaptor proteins, which associate with CRL2s, and CRL2^VHL^ polyubiquitinates and degrades HIF-1α subunits under normoxic conditions [60,61]. CRL2s also use RBX1 as the RING protein; therefore, our observed downregulation of RBX1 may also be lending itself to decreased CRL2^VHL^ function (Figure 1). Consequently, our observations of downregulated cullin-RING E3 ligase expression may be CRL4-specific or extend to E3 ligases in a broader sense, including (1) disincentivization of VHL-mediated polyubiquitination of HIF-1α during hypoxia by reducing CRL2^VHL^ or (2) Skp1/Skp2/RBX1 downregulation due to the CRL2^VHL^ complex no longer being necessary for HIF-1α degradation. Further research would need to be conducted into other families of E3 ligases and their expression/functional roles during hypoxia; however, we are confident in postulating that there is reduced functionality of the CRL4 complex architecture, with the possibility of this being extended to other CRLs (i.e., CRL2^VHL^) as well.

### 3.2. Hypoxia-Induced Upregulation of CYLD, USP9X, and DDB-1 May Link to DNA Damage Repair During Oxidative Stress

There was a marked upregulation in deubiquitinating enzymes CYLD and USP9X expression during hypoxia (Figure 2), which corroborates our results indicating decreased protein degradation through the UPS. USP9X can degrade VHL by protecting its substrate SMAD-specific E3 ubiquitin protein ligase 1 (SMURF1), to the extent that inhibiting USP9X suppresses HIF activity [71]. Given the constitutive expression of HIF-1α during both hypoxia and normoxia in *H. glaber* [34], allowing for mutations in both HIF-1α (T407 to I) and VHL (V166 to I) [72,73], other functional roles for hypoxia-elevated USP9X can be investigated. Along with robust expression of HIF-1α in multiple tissues [73], there is a corresponding upregulation with p21 and nuclear factor kappa B (NFκB) in hypoxic naked mole-rat brains [74]. NFκB activation is one of the first responses to hypoxia and well-characterized in *H. glaber* [75]. Among its other functions, VHL suppresses NFκB signaling through phosphorylation of caspase recruitment domain-containing protein 9 (CARD9), an NFκB agonist [76]. Increased expression of USP9X could therefore degrade VHL and promote NFκB activation as its primary function during hypoxia, in lieu of the obsolete need to stabilize HIF-1α by degrading VHL.

CYLD is a negative regulator of the NFκB pathway and c-Jun N-terminal kinase (JNK) signaling through nuclear factor-kappa B essential modulator (NEMO) and TNF receptor-associated factor 2 (TRAF2) [77]. Inactivation of CYLD causes NFκB activation and vice versa [78]; but there are circumstances in which CYLD can activate NFκB, such as association with spermatogenesis-associated 2 (SPATA2) during hypoxia-induced ER stress, which triggers pyroptic cell death through induction of the NLR family pyrin domain containing 3 (NLRP3) inflammasome [79]. CYLD is usually downregulated in hypoxic environments, as observed in many cancer cell lines [80]. Hypoxia enhances both basal and tumor necrosis factor alpha (TNFα)-induced expression of various proinflammatory cytokines, whereas CYLD overexpression strongly counteracts these responses [80,81]. Therefore, an increase in CYLD expression may be a detrimental response to hypoxia itself. AMPK, widely regarded as an energy sensor for the cell, is downregulated in hypoxic naked mole-rat skeletal muscle for tissue-specific reprioritization of energy [82]. While hypoxia normally triggers AMPK in rat cardiac myoblasts, artificially inhibiting AMPK caused dramatic augmentation in JNK activation, inflammatory NFκB phosphorylation, apoptosis, generation of ROS, and mitochondrial dysfunction during hypoxia and reoxygenation [83]—all of these factors are, in turn, inhibitors of CYLD [81,84]. Curiously, inhibiting JNK activation reduced NFκB phosphorylation and apoptosis without an effect on AMPK activation [83].

Differential modulation of inflammatory pathways, DNA damage repair, and NFκB signaling are hallmark characteristics in long-lived species such as *H. glaber* [85], with increased DNA damage repair gene transcription and efficient DNA repair correlating with longer life spans [86,87,88]. Liver RNA-seq analysis comparing *H. glaber* and mice revealed a higher transcription rate of DNA damage repair-associated genes across non-homologous end joining (NHEJ), mismatch repair (MMR), homology-directed repair (HDR), and base-excision repair (BER) pathways [86], with BER attenuating DNA damage far more rapidly in *H. glaber* than mice [89]. Moreover, naked mole-rats upregulate expression of UBE1, which catalyzes the first step of ubiquitin conjugation [90], and UBE2v2, which stimulates the DNA damage response by promoting K63-polyubiquitination of histones [91], over the course of their lifespan [92]. The UPS is deeply implicated in DNA damage, especially since ubiquitination/other E3 ligases are pivotal components in recognizing and activating DNA repair pathways. For example, DNA double-strand break (DSB) repair is initiated when histone H2AX and mediator of DNA damage checkpoint 1 (MDC1) are phosphorylated by ataxia-telangiectasia mutated (ATM), creating γH2AX and triggering recruitment of several E3 ligases [93,94]. Polycomb group proteins, which mediate transcriptional repression/chromatin silencing, aid recruitment of breast cancer-associated 1 (BRCA1; a RING-finger ubiquitin ligase that recognizes γH2AX) and TP53BP1 via H2A/H2AX ubiquitination [95]. HERC2, a large HECT-E3 ligase, associates with RNF8 to form the RNF8-UBC13 complex and aids deubiquitinating enzyme USP16 with fine-tuning ubiquitination during repair [96,97,98]. All of this can be linked to our results and the 2.5-fold upregulation of DDB-1 during hypoxia (Figure 1); DDB-1 was originally discovered for its function in nucleotide excision repair (NER) and recognizing UV-damaged DNA [99]. Given that DDB-1 is the unique adaptor protein to CUL4A, which was downregulated during hypoxia (Figure 1), it is reasonable to surmise that DDB-1/the UPS may be predominantly assisting with DNA damage repair from both longevity and oxidative stress perspectives during hypoxia, potentially supporting other antioxidant pathways such as NFκB.

### 3.3. Increased Proteasomal Activity May Link to Longevity as Well as Hypoxia Tolerance

A striking upregulation of all three metrics of proteasomal activity (caspase-like, chymotrypsin-like, and trypsin-like) in hypoxic *H. glaber* initially runs counterintuitive to our broadly observed downregulation of CRL proteins (Figure 1) and upregulation of DUBs (Figure 2), suggesting lower rates of protein degradation during hypoxia in the *H. glaber* heart. However, our results augment the existing literature for longevity in this species when compared to mice, an analogous species with a short lifespan. Juvenile naked mole-rats have higher basal levels of free protein thiol groups (1.6-fold) and native proteasome activity, as well as less protein ubiquitination and urea-induced protein unfolding [44]. Liver proteasomal activity in naked mole-rats (quantified through chymotrypsin-like, caspase-like, and trypsin-like assays) was double per µg protein compared to mice [45], and proteasomal activity increased 1.5-fold further over *H. glaber’s* lifespan via both ubiquitin-independent (facilitated by 20S proteasome) and ubiquitin-dependent degradation (facilitated by 26S proteasome) [45,100,101]. This increase in proteolytic activity was not attributed to *H. glaber* possessing higher total amounts of proteasome; naked mole-rats and mice had similar total 26S proteasome and 20S proteasomal units (with 26S proteasome more populous), yet 26S specific activity was 12-fold higher and 20S specific activity was 4-fold higher in *H. glaber* compared to mice [45]. However, both 19S subunits and immunoproteasome catalytic subunits were elevated in naked mole-rats, suggesting a greater proportion of immunoproteasomes in juvenile livers to “prime” adults for efficient removal of stress-damaged proteins as one potential contributor for increased proteasomal activity [45].

Higher levels of native proteasomal activity and protein degradation/turnover have been proposed as hypotheses for the extreme longevity of naked mole-rats, combatting the increased buildup of aberrant proteins and cytotoxic byproducts traditionally correlated with age (as encapsulated by the oxidative stress theory of aging) [6,102,103]. However, the overlap between oxidative stress from an aging perspective and oxidative stress as an innate byproduct of hypoxia may be difficult to untangle—especially given the resilience of *H. glaber* to oxygen fluctuation—and leading to a “chicken-and-egg” issue for adaptations straddling longevity and hypoxia tolerance. For example, hypoxia can lead to altered cellular localization and activity of the proteasome without changing the total cellular quantity of the proteosome itself; in cancer cells, hypoxia induction led to a 2-fold increase in proteasomal translocation to the nucleus, with an associated 2-fold increase in proteasomal activity [104].

A subset of the oxidative stress theory of aging specifically focuses on mitochondria and their role in mitigating ROS balance/accumulation [105,106,107,108]. The RBR-E3 ubiquitin ligase parkin (PRKN) is largely responsible for linking mitophagy with the UPS; however, damaged mitochondria are removed by PRKN-independent mitophagy following hypoxia-induced oxidative stress [109]. Hypoxic mitochondria are heavily K48-linked ubiquitinated, with proteins mitofusion 1/2 (MFN1/2) and outer membrane translocase 20 (TOM20) showing the most extensive degradation [109]. Therefore, during hypoxia and correlated oxidative stress, the UPS and receptor-mediated autophagy are responsible for clearing oxidation-damaged mitochondria [109,110,111]. *H. glaber* possesses unique mitochondrial dynamics throughout its lifespan [112,113,114], with underdeveloped mitochondria in skeletal muscle and cardiac muscle up to the age of 5 years, before developing an unusually robust mitochondrial structure that remains in place up to the 11 year mark with very little change and/or degradation [112]. These adaptations and delayed onset of age-related changes to mitochondrial structure include the age-related loss of muscle fibers (sarcopenia), as naked mole-rats have an altered Complex IV of mitochondria, which may be attributed to the maintenance of skeletal/cardiac muscle function for abnormally long timespans [38,115,116,117]. Skeletal muscle mass is a balance between rates of protein synthesis and protein degradation, which are often dysregulated during hypoxia [118,119,120]; a study demonstrating a 5-fold increase in protein degradation during chronic hypobaric hypoxia vastly outpacing the 1.5-fold rise in protein synthesis [118]. This protein degradation was attributed to the UPS, with increases in chymotrypsin-like activity and calpains [118].

Antioxidant pathways also demonstrate considerable overlap with longevity adaptations. Protein expression levels of NFκB and TNFα were markedly higher in naked mole-rat liver, and nuclear factor erythroid 2-related factor (Nrf2) demonstrated a 2.5-fold higher transcription rate and resultant 3–10-fold increase in protein expression in non-stressed naked mole-rats compared to mice [45,121,122]. BRCA1, previously mentioned for its role in DNA damage repair, displays positive selection in a region intrinsic to Nrf2 stability and diminished Keap1-induced ubiquitination/degradation of Nrf2 [123], suggesting a selective advantage for high basal expression of antioxidant genes in *H. glaber*. Nrf2 was identified as a critical indicator for maximum lifespan potential in a comparative study between naked mole-rats and nine other rodent species—naked mole-rat longevity was linked not to the protein levels of Nrf2 itself, but rather to a significant negative relationship with the regulators Keap1 and β-TrCP, which target Nrf2 for degradation [122]. This is both linked with our results of hypoxic downregulation of β-TrCP (Figure 1) and those of other studies documenting a 3-fold reduction in Keap1 transcription in *H. glaber* compared to mice [122]. Naked mole-rat brains also display age-related increases in heat shock proteins (HSPs) [92], which may synergize with the elevated protein flux through the proteasome to prevent the build-up of dysfunctional proteins [124]. Research in other anoxia-tolerant models suggests that levels of HSPs in the heart may be constitutively elevated and therefore warrant no further increase when anoxia is imposed, supported by elevated levels of myocardial HSP60 in turtles compared with anoxia-sensitive animals [125]. Taken together, further research to disentangle the hypoxia- and longevity-specific contributors of increased proteasomal activity with protein ubiquitination and antioxidant responses will be needed for *H. glaber*.

## 4. Materials and Methods

### 4.1. Animal Collection

Adult subordinate, non-breeding naked mole-rats (males and females) were used in this study from a group-housing facility at the University of Ottawa (Canada). Detailed procedures for animal feeding, monitoring, and behavior can be located in [37]. Naked mole-rats were randomly separated into two distinct experimental chambers (*n* = 5) containing a thin layer of bedding on the floor, then the chambers were sealed, and animals were acclimatized for 1 h prior to experimentation using an FC-10 O_2_ analyzer (Sable Systems International, Las Vegas, NV, USA) for continuous monitoring. Chambers were constantly ventilated with gas mixtures via calibrated rotameters, and inflowing gas was provided at a flow rate of 0.5 L/min, as assessed by a calibrated mass flow meter (Alicat Scientific, Tucson, AZ, USA). Normoxia was defined by a fractional gas composition of 20.95% O_2_, 0.05% CO_2_, and balance N_2_; and hypoxia exposure at 7% O_2_ was performed for 24 h (7% O_2_, 0.05% CO_2_, balance N_2_). Animals (*n* = 5 for each condition) were euthanized by conscious cervical dislocation, immediately followed by decapitation. Whole hearts were rapidly dissected within 30 s, flash frozen in liquid nitrogen, and stored at −80 °C until analysis.

All naked mole-rat experiments complied with the requirements of the Canadian Council on Animal Care and were approved by the University of Ottawa Animal Care Committee (protocol #2535).

### 4.2. Total Protein Isolation

To prepare total protein homogenates, ~50 mg samples of frozen cardiac tissue from normoxic and 24 h hypoxic *H. glaber* (*n* = 5) were crushed under liquid nitrogen and transferred to test tubes on ice. Samples were homogenized in 1:2.5 *w*/*v* homogenization buffer (20 mM HEPES, pH 7.5, 200 mM NaCl, 0.1 mM EDTA, 10 mM NaF, 1 mM Na_3_VO_4_, 15 mM β-glycerophosphate) along with several crystals of phenylmethylsulfonyl fluoride (PMSF) and 10 μL/mL of Sigma Protease Inhibitor. Homogenates were centrifuged at 4 °C for 15 min at 10,000× *g*, and supernatants were collected and transferred to sterile 1.5 mL microcentrifuge tubes. Protein concentrations were ascertained from the BioRad Protein Assay, with bovine serum albumin as the standard. Absorbance readings were measured at 595 nm on a Bio-Tek Power Wave HT Spectrophotometer using Gen5 software (Agilent BioTek, Winooski, VT, USA). To prepare samples for Western immunoblotting, protein concentrations were standardized and mixed 1:1 *v*:*v* with 2× SDS loading buffer (100 mM Tris-base, 4% *w*:*v* SDS, 20% *v*:*v* glycerol, 0.2% *w*:*v* bromophenol blue, 10% *v*:*v* 2-mercaptoethanol). Samples were boiled for 10 min at 100 °C to denature and linearize proteins and immediately transferred to ice. Samples were stored at −40 °C until use.

### 4.3. Western Immunoblotting

Prepared homogenates containing 20–25 μg of protein were loaded onto 8–15% SDS polyacrylamide gels (individual gel percentage dependent on the molecular weight of the target protein). Gels were composed of 8–15% *v*/*v* acrylamide, 130 mM Tris buffer (pH 6.8 for stacking gel and pH 8.8 for resolving gel), 0.1% SDS, 0.1% ammonium persulfate, and 0.1% TEMED. Proteins were separated via electrophoresis for 45–130 min at 180 V in Tris–glycine running buffer (stock buffer contained 75.5 g Tris-base, 460 g glycine, 25 g SDS, with ddH_2_O to a total of 2.5 L) using a BioRad Mini-Protean 3 System. Aliquots of 5.5 μL of pre-stained protein molecular weight ladders were run concurrently with the samples to serve as a molecular weight reference.

Following electrophoresis, proteins were electroblotted by wet transfer onto 0.45 μm PVDF membranes in transfer solution (25 mM Tris pH 8.8, 192 mM glycine, and 10% *v*/*v* methanol) at 4 °C for 2 h at 160 mA using BioRad Mini-Protean Transfer cells (Bio-Rad, Hercules, CA, USA). After transfer, PVDF membranes were blocked with 10% milk for 30 min before washing 3 × 5 min in TBST (10 mM Tris, 150 mM NaCl, 0.05% *v*/*v* Tween-20, pH 7.5). Membranes were incubated with 1:1000 *v*:*v* primary antibody overnight at 4 °C.

Following overnight incubation with the primary antibody, all PVDF membranes were washed 4 × 5 min in TBST. Membranes were incubated in 1:5000 *v*:*v* HRP-conjugated secondary antibody (anti-rabbit; Bioshop; Cat. # APA007P) diluted in TBST for 30 min. Membranes were washed 4 × 5 min with TBST and visualized using chemiluminescence (1.4 mL of luminol and H_2_O_2_ in a 1:1 ratio) in a ChemiGenius Bio-Imaging System (Syngene, Frederick, MD, USA) with quantification of band densities using the GeneSnap software. Membranes were stained with Coomassie blue (0.25% *w*:*v* Coomassie brilliant blue, 7.5% *v*:*v* acetic acid, 50% *v*:*v* methanol) for use as loading controls via total protein analysis, with individual protein band densities quantified with GeneSnap on the ChemiGenius Bio-Imaging System using normal light settings to image the blue bands.

The antibodies used were purchased in a Ubiquitin E3 Ligase Complex Antibody Sampler Kit (Cell Signaling; Cat. #: 12724, Cell Signaling, Danvers, MA, USA), which included the specific targets as follows: CUL4A; CYLD; DDB-1; DDB-2; RBX1; Skp1; Skp2; and β-TrCP. Targets were also selected from a DUB Antibody Sampler Kit (Cell Signaling; Cat #: 8353) and included the following: phospho-CYLD (Ser418); STAMBP; A20/TNFAIP3; UCHL1; HAUSP; USP9X; UCHL3; and USP10.

### 4.4. Preparation of Reagents and Samples for Proteasomal Activity Assay

Proteasomal activity was measured with a commercially available Proteasome-Glo^TM^ 3-Substrate System Assay, which measures caspase-like, chymotrypsin-like, and trypsin-like activities in a luminescent system. Broadly, the Luciferin Detection Reagent, Proteasome-Glo^TM^ Buffer, and Proteasome-Glo^TM^ Substrates were equilibrated to room temperature before preparation. The Luciferin Detection Reagent was reconstituted with 10 mL Proteasome-Glo^TM^ Buffer, and the solution was gently vortexed until there was no precipitate (~1 min). The appropriate substrate (caspase-like assay, ZnLPnLD-Glo^TM^; chymotrypsin-like assay, Suc-LLVY-Glo^TM^; trypsin-like assay, Z-LRR-Glo^TM^) was added to their respective reconstituted Luciferin Detection Reagent for a final concentration of 40 µM in solution. These three prepared Proteasome-Glo^TM^ Reagents were incubated for 1 h at room temperature to remove trace contamination of free aminoluciferin and stored at 4 °C until use.

Crude protein extracts for assaying proteasomal activity were replicated from the procedure in [126]. Briefly, 100 mg of flash-frozen cardiac tissue from normoxic vs. 24 h hypoxic *H. glaber* (*n* = 4) was crushed under liquid nitrogen and transferred to 2 mL microcentrifuge tubes. Homogenization buffer (50 mM Tris-HCl, pH 7.8; 10 mM β-mercaptoethanol; 5 mM EDTA; 5 mM EGTA; 50 mM NaF) was added to samples in 1:10 *w*/*v*, and tissue was homogenized with a Polytron homogenizer. Samples were incubated for 5 min on ice and were centrifuged at 9000× *g* for 15 min at 4 °C. The protein-containing supernatant was transferred to a sterile 1.5 mL microcentrifuge tube, and protein concentration was determined using the BioRad Protein Assay with bovine serum albumin as the standard. Absorbance readings were measured at 595 nm on a Bio-Tek Power Wave HT Spectrophotometer using Gen5 software. Samples were standardized to 5 µg/µL using homogenization buffer and stored at −80 °C until use.

### 4.5. Proteasomal Activity Assay

Working concentration was optimized to 50 µg of protein using a 1:2 serial dilution series of pooled crude protein extracts, and assay incubation length was determined at 10 min and image exposure at 2 min to prevent saturation and overexposure of wells (for standard curve, see Figure S1). The experimental 96-well opaque black microplate was set up with *n* = 4 of control vs. stressed samples for all three assays (caspase-like, chymotrypsin-like, and trypsin-like). Samples were adjusted to 50 µL with homogenization buffer and added to wells with 50 µL of their respective Proteasome-Glo^TM^ Reagent for a final reaction volume of 100 µL. Microplates were incubated for 10 min at room temperature, and luminescence was quantified on a Chemi-Genius Bio-Imaging System (Syngene, Frederick, MD, USA) according to the method laid out in [127].

### 4.6. Data Quantification and Statistics

For Western immunoblotting, variability in protein loading across each gel was ensured by normalizing PVDF band intensities of the target of interest against the total intensity of a group of Coomassie-stained protein bands (excluding the target protein) in the same lane, demonstrating constant expression across the experimental replicates [128]. Final analysis utilized *n* = 4 or *n* = 5, adjusted for experimental concerns including the following: outliers; abnormal protein degradation in a singular lane; improper secondary antibody binding; membrane damage; and transfer anomalies; etc.

Well-scaling factors for the proteasomal activity assay were calculated from [127] by incubating 100 µL per well of a 2:1 *v*:*v* mixture of chemiluminescent solution (1.25 mM luminol, 200 μM p-coumaric acid, 1:3000 *v*:*v* H_2_O_2_ and 100 mM Tris buffer pH 8.8) and HRP-conjugated anti-rabbit antibody (1:8000 *v*:*v* in 0.05% TBST) for 2 min on the same experimental microplate and replicating the position of the microplate and imaging settings in the Chemi–Genius system. The densitometric raw volume of every well was quantified and normalized to well A1 (upper-left) of the entire plate, resulting in a scaling factor for each well. The densitometric value of each experimental data point was divided by the well scaling factor to correct for signal variation based on the angle of incidence relative to the camera. The heatmap of well scaling factors can be found in Figure S2.

All data are reported as mean ± SEM (*n* = 4–5), with experimental conditions reported relative to controls, which have been standardized to 1. Densitometry and background correction were performed with GeneTools software (V.4.3.8.0) (Syngene; https://www.syngene.com/support/software, accessed on 30 May 2025), while RBioPlot [129] was used for histogram generation and testing for significant differences (control vs. experimental) using a Student’s *t*-test, with *p* < 0.05 accepted as a significant difference.

## 5. Conclusions

Overall, we observed downregulation of CRL4 architecture in cardiac tissue of normoxic vs. 24 h hypoxic *H. glaber.* Overexpression of DDB-1 during stress pointed to a direct role in DNA damage repair, which is in itself heavily reliant on the UPS and histone ubiquitination. Relative expression of deubiquitinating enzymes USP9X and CYLD was upregulated, with analogous molecular rationale involving oxidative stress and the HIF-1-VHL-NFκB axis. Moreover, the proposed increase in hypoxia-induced NFκB and Nrf antioxidant defenses coupled with naked mole-rats’ elevated innate HIF-1α and VEGF expression to counter ROS has strong links to human models of hypoxic injury [130,131]. HIF signaling transduction via changes in erythropoietin or VEGF can lead to adjustments of the oxygen-carrying capacity of the blood (e.g., increased erythropoiesis) and enhanced vascularization [132]; for example, development of high-altitude polycythemia (HAPC) in the gastric mucosa is characterized by increased capillary density/erythropoiesis as well as elevations in ROS generation, HIF-1α, apoptosis, and mitochondrial vacuole density [133]. Likewise, susceptibility to high-altitude pulmonary edema (HAPE) is linked to differential expression of HIF-1α, prolyl hydroxylase domain 1 (PHD1), PHD3, pyruvate dehydrogenase kinase 1 (PDK1), mitochondrial transcription factor A (TFAM), peroxisome proliferator-activated receptor gamma coactivator 1 alpha (PPARGC1α), and NRF1 [134]. Development of HAPE was also associated with mitochondrial mutations in Complexes I–V and disruption of subunit assembly/proton pumping activity [134]—providing an intriguing avenue for naked mole-rats’ resilient mitochondrial dynamics/structural adaptations to inform countermeasures for human hypoxia-induced mitochondrial damage, fluctuations in mitochondrial mass and dynamics, and increased mitochondrial apoptosis [135,136,137,138]. Indeed, certain human populations that have acclimatized to high-altitude living (such as Tibetan sherpas) exhibit molecular characteristics similar to those associated with naked mole-rat hypoxia tolerance. Sherpas display attenuated levels of both oxidative stress while also maintaining muscle ATP and phosphocreatine levels in the face of decreased oxygen delivery [139]. Some populations have increased variants of a transcriptional regulator of fatty acid metabolism, peroxisome proliferator-activated receptor alpha (PPARα)—which may potentially improve the efficiency of oxygen utilization by lowering capacity for fatty acid oxidation and reducing the accumulation of intramuscular lipid intermediates at high altitudes [139].

Sherpas also possess adaptive gene variants within the HIF pathway to combat erythropoiesis and increased blood lactate, again analogous to *H. glaber’s* unique metabolic profile allowing it to not just resist blood lactate accumulation but use lactate to inhibit aerobic metabolism in cardiac mitochondria [31]. Oxidative stress may also contribute to our observed elevation of proteasomal activity in *H. glaber*, which conceivably links to longevity and the oxidative stress theory of aging for this particular species.

Taken together, this study did not highlight any particular importance for CRL4 function during extreme environmental stress but provided a unique outlook on proteasomal function and potential UPS integration with oxidative stress, longevity, and hypoxia.

## Data Availability

Data is contained within the article or Supplementary Material. Dataset available on request from the authors.

## Supplementary Materials

The following supporting information can be downloaded at: https://www.mdpi.com/article/10.3390/muscles5010006/s1, Figure S1: Standard curve for proteasomal assay; Figure S2: Well-scaling factors for proteasomal assay; Table S1: Raw data for proteasomal activity assay.

## Abbreviations

The following abbreviations are used in this manuscript:

AMPK	AMP-activated protein kinase
β-TrCP	beta-transducin repeat-containing E3 ubiquitin protein ligase
CARD9	caspase recruitment domain-containing protein 9
Cdt	cytolethal distending toxin
CRL	cullin-RING E3 ligase
CUL	cullin
CYLD	cylindromatosis lysine 63 deubiquitinase
DDB	DNA damage binding
DNA	deoxyribonucleic acid
DUB	deubiquitinating enzyme
EDTA	ethylenediaminetetraacetic acid
EGTA	ethylene glycol-bis(β-aminoethyl ether)-N,N,N′,N′-tetraacetic acid; egtazic acid
EloBC	elongin BC
ER	endoplasmic reticulum
HAPC	high-altitude polychythemia
HAPE	high-altitude pulmonary edema
HAUSP	herpesvirus-associated ubiquitin-specific protease
HECT	homology to E6-associated protein C-terminus
HEPES	4-(2-hydroxyethyl)-1-piperazineethanesulfonic acid
HIF	hypoxia inducible factor
HRP	horseradish peroxidase
JNK	c-Jun N-terminal kinase
Keap	Kelch-like ECH-associated protein
MFN	mitofusion
MRD	metabolic rate depression
mTORC1	mammalian/mechanistic target of rapamycin complex 1
NEMO	nuclear factor-kappa B essential modulator
NFκB	nuclear factor kappa B
NLRP	NLR family pyrin domain-containing
Nrf2	nuclear factor erythroid 2-related factor
PHD	prolyl hydroxylase domain
PDK1	pyruvate dehydrogenase kinase 1
PPAR	peroxisome proliferator-activated receptor
PRKN	parkin
PVDF	polyvinylidene fluoride
RBR	RING-between-RING
RBX	RING box protein
RING	really interesting new gene
RNA	ribonucleic acid
ROS	reactive oxygen species
SDS	sodium dodecyl sulfate
SEM	standard error of the mean
Skp	seventeen kilodalton protein
SMURF1	SMAD-specific E3 ubiquitin protein ligase 1
SPATA2	spermatogenesis associated 2
STAMBP	STAM binding protein gene
SUMO	small ubiquitin-like modifier
TBST	Tris-buffered saline and Tween-20
TEMED	tetramethylethyldiamine
TNFα	tumor necrosis factor alpha
TNFAIP3	TNF alpha induced protein 3
TOM20	outer membrane translocase 20
TRAF2	TNF receptor-associated factor
UCHL	ubiquitin C-terminal hydrolase
UPS	ubiquitin–proteasome system
USP	ubiquitin-specific protease
VHL	von Hippel–Lindau

## References

[1] McGarry T., Biniecka M., Veale D.J., Fearon U. (2018). Hypoxia, Oxidative Stress and Inflammation. Free Radic. Biol. Med..

[2] Bae T., Hallis S.P., Kwak M.K. (2024). Hypoxia, Oxidative Stress, and the Interplay of HIFs and NRF2 Signaling in Cancer. Exp. Mol. Med..

[3] Islam M.T. (2017). Oxidative Stress and Mitochondrial Dysfunction-Linked Neurodegenerative Disorders. Neurol. Res..

[4] Reddan B., Cummins E.P. (2025). The Regulation of Cell Metabolism by Hypoxia and Hypercapnia. J. Biol. Chem..

[5] Athmuri D.N., Bhattacharyya J., Bhatnagar N., Shiekh P.A. (2024). Alleviating Hypoxia and Oxidative Stress for Treatment of Cardiovascular Diseases: A Biomaterials Perspective. J. Mater. Chem. B.

[6] Lewis K.N., Andziak B., Yang T., Buffenstein R. (2013). The Naked Mole-Rat Response to Oxidative Stress: Just Deal with It. Antioxid. Redox Signal..

[7] Hermes-Lima M., Zenteno-Savín T. (2002). Animal Response to Drastic Changes in Oxygen Availability and Physiological Oxidative Stress. Comp. Biochem. Physiol. C Toxicol. Pharmacol..

[8] Hochachka P.W., Buck L.T., Doll C.J., Land S.C. (1996). Unifying Theory of Hypoxia Tolerance: Molecular/Metabolic Defense and Rescue Mechanisms for Surviving Oxygen Lack. Proc. Natl. Acad. Sci. USA.

[9] Jiang M., Fan X., Wang Y., Sun X. (2023). Effects of Hypoxia in Cardiac Metabolic Remodeling and Heart Failure. Exp. Cell Res..

[10] Zhang L. (2005). Prenatal Hypoxia and Cardiac Programming. J. Soc. Gynecol. Investig..

[11] Frasch M.G., Giussani D.A. (2020). Impact of Chronic Fetal Hypoxia and Inflammation on Cardiac Pacemaker Cell Development. Cells.

[12] Wu J., Stefaniak J., Hafner C., Schramel J.P., Kaun C., Wojta J., Ullrich R., Tretter V.E., Markstaller K., Klein K.U. (2016). Intermittent Hypoxia Causes Inflammation and Injury to Human Adult Cardiac Myocytes. Anesth. Analg..

[13] de Theije C., Costes F., Langen R.C., Pison C., Gosker H.R. (2011). Hypoxia and Muscle Maintenance Regulation: Implications for Chronic Respiratory Disease. Curr. Opin. Clin. Nutr. Metab. Care.

[14] Nguyen T.H., Conotte S., Belayew A., Declèves A.E., Legrand A., Tassin A. (2021). Hypoxia and Hypoxia-Inducible Factor Signaling in Muscular Dystrophies: Cause and Consequences. Int. J. Mol. Sci..

[15] Sekar J., Attaway A.H. (2023). The Intersection of HIF-1α, O-GlcNAc, and Skeletal Muscle Loss in Chronic Obstructive Pulmonary Disease. Glycobiology.

[16] Langen R.C.J., Gosker H.R., Remels A.H.V., Schols A.M.W.J. (2013). Triggers and Mechanisms of Skeletal Muscle Wasting in Chronic Obstructive Pulmonary Disease. Int. J. Biochem. Cell Biol..

[17] Pasiakos S.M., Berryman C.E., Carrigan C.T., Young A.J., Carbone J.W. (2017). Muscle Protein Turnover and the Molecular Regulation of Muscle Mass during Hypoxia. Med. Sci. Sports Exerc..

[18] Valle-Tenney R., Rebolledo D., Acuña M.J., Brandan E. (2020). HIF-Hypoxia Signaling in Skeletal Muscle Physiology and Fibrosis. J. Cell Commun. Signal..

[19] Chaillou T., Koulmann N., Meunier A., Pugnière P., McCarthy J.J., Beaudry M., Bigard X. (2014). Ambient Hypoxia Enhances the Loss of Muscle Mass after Extensive Injury. Pflug. Arch..

[20] Brett R.A. (1986). The Ecology and Behaviour of the Naked Mole-Rat, *Heterocephalus glaber* Ruppell (Rodenti: Bathyergidae). Ph.D. Thesis.

[21] Buffenstein R., Amoroso V., Andziak B., Avdieiev S., Azpurua J., Barker A.J., Bennett N.C., Brieño-Enríquez M.A., Bronner G.N., Coen C. (2022). The Naked Truth: A Comprehensive Clarification and Classification of Current ‘Myths’ in Naked Mole-rat Biology. Biol. Rev. Camb. Philos. Soc..

[22] Shams I., Avivi A., Nevo E. (2005). Oxygen and Carbon Dioxide Fluctuations in Burrows of Subterranean Blind Mole Rats Indicate Tolerance to Hypoxic-Hypercapnic Stresses. Comp. Biochem. Physiol. A Mol. Integr. Physiol..

[23] Browe B.M., Vice E.N., Park T.J. (2020). Naked Mole-Rats: Blind, Naked, and Feeling No Pain. Anat. Rec..

[24] Reznick J., Park T.J., Lewin G.R. (2021). A Sweet Story of Metabolic Innovation in the Naked Mole-Rat. Adv. Exp. Med. Biol..

[25] Park T.J., Smith E.S.J., Reznick J., Bennett N.C., Applegate D.T., Larson J., Lewin G.R. (2021). African Naked Mole-Rats Demonstrate Extreme Tolerance to Hypoxia and Hypercapnia. Adv. Exp. Med. Biol..

[26] Pamenter M.E. (2022). Adaptations to a Hypoxic Lifestyle in Naked Mole-Rats. J. Exp. Biol..

[27] Widmer H.R., Hoppeler H., Nevo E., Taylor C.R., Weibel E.R. (1997). Working Underground: Respiratory Adaptations in the Blind Mole Rat. Proc. Natl. Acad. Sci. USA.

[28] Shams I., Avivi A., Nevo E. (2004). Hypoxic Stress Tolerance of the Blind Subterranean Mole Rat: Expression of Erythropoietin and Hypoxia-Inducible Factor 1α. Proc. Natl. Acad. Sci. USA.

[29] Storey K.B., Storey J.M. (1990). Metabolic Rate Depression and Biochemical Adaptation in Anaerobiosis, Hibernation and Estivation. Q. Rev. Biol..

[30] Storey K.B., Storey J.M. (2004). Metabolic Rate Depression in Animals: Transcriptional and Translational Controls. Biol. Rev. Camb. Philos. Soc..

[31] Pamenter M.E., Dzal Y.A., Thompson W.A., Milsom W.K. (2019). Do Naked Mole Rats Accumulate a Metabolic Acidosis or an Oxygen Debt in Severe Hypoxia?. J. Exp. Biol..

[32] Park T.J., Reznick J., Peterson B.L., Blass G., Omerbašić D., Bennett N.C., Kuich P.H.J.L., Zasada C., Browe B.M., Hamann W. (2017). Fructose-Driven Glycolysis Supports Anoxia Resistance in the Naked Mole-Rat. Science.

[33] Ojaghi M., Pamenter M.E. (2024). Hypoxia Impairs Blood Glucose Homeostasis in Naked Mole-Rat Adult Subordinates but Not Queens. J. Exp. Biol..

[34] Faulkes C.G., Eykyn T.R., Miljkovic J.L., Gilbert J.D., Charles R.L., Prag H.A., Patel N., Hart D.W., Murphy M.P., Bennett N.C. (2024). Naked Mole-Rats Have Distinctive Cardiometabolic and Genetic Adaptations to Their Underground Low-Oxygen Lifestyles. Nat. Commun..

[35] Yang Z.J., Zhang W.F., Jin Q.Q., Wu Z.R., Du Y.Y., Shi H., Qu Z.S., Han X.J., Jiang L.P. (2024). Lactate Contributes to Remote Ischemic Preconditioning–Mediated Protection Against Myocardial Ischemia Reperfusion Injury by Facilitating Autophagy via the AMP-Activated Protein Kinase–Mammalian Target of Rapamycin–Transcription Factor EB–Connexin 43 Axis. Am. J. Pathol..

[36] Kowalski D.P., Aw T.Y., Park Y., Jones D.P. (1992). Postanoxic Oxidative Injury in Rat Hepatocytes: Lactate-Dependent Protection against Tert-Butylhydroperoxide. Free Radic. Biol. Med..

[37] Houlahan C.R., Kirby A.M., Dzal Y.A., Fairman G.D., Pamenter M.E. (2018). Divergent Behavioural Responses to Acute Hypoxia between Individuals and Groups of Naked Mole Rats. Comp. Biochem. Physiol. B Biochem. Mol. Biol..

[38] Holtze S., Eldarov C.M., Vays V.B., Vangeli I.M., Vysokikh M.Y., Bakeeva L.E., Skulachev V.P., Hildebrandt T.B. (2016). Study of Age-Dependent Structural and Functional Changes of Mitochondria in Skeletal Muscles and Heart of Naked Mole Rats (Heterocephalus Glaber). Biochemistry.

[39] Band M., Joel A., Hernandez A., Avivi A. (2009). Hypoxia-induced BNIP3 Expression and Mitophagy: In Vivo Comparison of the Rat and the Hypoxia-tolerant Mole Rat, Spalax Ehrenbergi. FASEB J..

[40] Nakada Y., Canseco D.C., Thet S., Abdisalaam S., Asaithamby A., Santos C.X., Shah A.M., Zhang H., Faber J.E., Kinter M.T. (2017). Hypoxia Induces Heart Regeneration in Adult Mice. Nature.

[41] Wang J.Z., Zhang Y.H., Du W.T., Liu G., Zhang X.Y., Cheng S.Z., Guo X.H. (2019). A Post-Surgical Adjunctive Hypoxic Therapy for Myocardial Infarction: Initiate Endogenous Cardiomyocyte Proliferation in Adults. Med. Hypotheses.

[42] Kimura W., Nakada Y., Sadek H.A. (2017). Hypoxia-Induced Myocardial Regeneration. J. Appl. Physiol..

[43] Rochette L., Malka G., Cottin Y. (2017). Hypoxia and Heart Regeneration: A New Paradoxical Approach for Cardioprotection. Arch. Cardiovasc. Dis..

[44] Pérez V.I., Buffenstein R., Masamsetti V., Leonard S., Salmon A.B., Mele J., Andziak B., Yang T., Edrey Y., Friguet B. (2009). Protein Stability and Resistance to Oxidative Stress Are Determinants of Longevity in the Longest-Living Rodent, the Naked Mole-Rat. Proc. Natl. Acad. Sci. USA.

[45] Rodriguez K.A., Edrey Y.H., Osmulski P., Gaczynska M., Buffenstein R. (2012). Altered Composition of Liver Proteasome Assemblies Contributes to Enhanced Proteasome Activity in the Exceptionally Long-Lived Naked Mole-Rat. PLoS ONE.

[46] Rodriguez K.A., Valentine J.M., Kramer D.A., Gelfond J.A., Kristan D.M., Nevo E., Buffenstein R. (2016). Determinants of Rodent Longevity in the Chaperone-Protein Degradation Network. Cell Stress Chaperones.

[47] Oka K., Yamakawa M., Kawamura Y., Kutsukake N., Miura K. (2023). The Naked Mole-Rat as a Model for Healthy Aging. Annu. Rev. Anim. Biosci..

[48] Al-attar R., Childers C.L., Nguyen V.C., Pamenter M.E., Storey K.B. (2020). Differential Protein Phosphorylation Is Responsible for Hypoxia-Induced Regulation of the Akt/MTOR Pathway in Naked Mole Rats. Comp. Biochem. Physiol. A Mol. Integr. Physiol..

[49] Orlowski M., Wilk S. (2000). Catalytic Activities of the 20 S Proteasome, a Multicatalytic Proteinase Complex. Arch. Biochem. Biophys..

[50] Neutzner A., Benard G., Youle R.J., Karbowski M. (2008). Role of the Ubiquitin Conjugation System in the Maintenance of Mitochondrial Homeostasis. Ann. N. Y. Acad. Sci..

[51] Neutzner M., Neutzner A. (2012). Enzymes of Ubiquitination and Deubiquitination. Essays Biochem..

[52] Dósa A., Csizmadia T. (2022). The Role of K63-Linked Polyubiquitin in Several Types of Autophagy. Biol. Futur..

[53] Pickart C.M., Fushman D. (2004). Polyubiquitin Chains: Polymeric Protein Signals. Curr. Opin. Chem. Biol..

[54] Urbé S. (2005). Ubiquitin and Endocytic Protein Sorting. Essays Biochem..

[55] Wang Y.T., Liu T.Y., Shen C.H., Lin S.Y., Hung C.C., Hsu L.C., Chen G.C. (2022). K48/K63-Linked Polyubiquitination of ATG9A by TRAF6 E3 Ligase Regulates Oxidative Stress-Induced Autophagy. Cell Rep..

[56] Varadan R., Assfalg M., Haririnia A., Raasi S., Pickart C., Fushman D. (2004). Solution Conformation of Lys63-Linked Di-Ubiquitin Chain Provides Clues to Functional Diversity of Polyubiquitin Signaling. J. Biol. Chem..

[57] Nandi D., Tahiliani P., Kumar A., Chandu D. (2006). The Ubiquitin-Proteasome System. J. Biosci..

[58] Petroski M.D., Deshaies R.J. (2005). Function and Regulation of Cullin-RING Ubiquitin Ligases. Nat. Rev. Mol. Cell Biol..

[59] Soucy T.A., Smith P.G., Rolfe M. (2009). Targeting NEDD8-Activated Cullin-RING Ligases for the Treatment of Cancer. Clin. Cancer Res..

[60] Nguyen H.C., Wang W., Xiong Y. (2017). Cullin-RING E3 Ubiquitin Ligases: Bridges to Destruction. Subcell. Biochem..

[61] Sarikas A., Hartmann T., Pan Z.Q. (2011). The Cullin Protein Family. Genome Biol..

[62] Diehl C.J., Ciulli A. (2022). Discovery of Small Molecule Ligands for the von Hippel-Lindau (VHL) E3 Ligase and Their Use as Inhibitors and PROTAC Degraders. Chem. Soc. Rev..

[63] Maxwell P.H., Wlesener M.S., Chang G.W., Clifford S.C., Vaux E.C., Cockman M.E., Wykoff C.C., Pugh C.W., Maher E.R., Ratcliffe P.J. (1999). The Tumour Suppressor Protein VHL Targets Hypoxia-Inducible Factors for Oxygen-Dependent Proteolysis. Nature.

[64] Cockman M.E., Masson N., Mole D.R., Jaakkola P., Chang G.W., Clifford S.C., Maher E.R., Pugh C.W., Ratcliffe P.J., Maxwell P.H. (2000). Hypoxia Inducible Factor-Alpha Binding and Ubiquitylation by the von Hippel-Lindau Tumor Suppressor Protein. J. Biol. Chem..

[65] Kang J.S., Kim D., Rhee J., Seo J.Y., Park I., Kim J.H., Lee D., Lee W.U., Kim Y.L., Yoo K. (2023). Baf155 Regulates Skeletal Muscle Metabolism via HIF-1a Signaling. PLoS Biol..

[66] Wlodarczyk J., Leng A., Abadchi S.N., Shababi N., Mokhtari-Esbuie F., Gheshlaghi S., Ravari M.R., Pippenger E.K., Afrasiabi A., Ha J. (2024). Transfection of Hypoxia-Inducible Factor-1α MRNA Upregulates the Expression of Genes Encoding Angiogenic Growth Factors. Sci. Rep..

[67] Willmore W.G., Storey K.B. (1996). Multicatalytic Proteinase Activity in Turtle Liver: Responses to Anoxia Stress and Recovery. Biochem. Mol. Biol. Int..

[68] Kulikov V.P., Tregub P.P., Osipov I.S., Trukhanov A.I. (2019). Hypercapnic Hypoxia as a Potential Means to Extend Life Expectancy and Improve Physiological Activity in Mice. Biogerontology.

[69] Tregub P.P., Komleva Y.K., Kulikov V.P., Chekulaev P.A., Tregub O.F., Maltseva L.D., Manasova Z.S., Popova I.A., Andriutsa N.S., Samburova N.V. (2024). Relationship between Hypoxia and Hypercapnia Tolerance and Life Expectancy. Int. J. Mol. Sci..

[70] Di Gregorio J., Cilenti L., Ambivero C.T., Andl T., Liao R., Zervos A.S. (2021). UBXN7 Cofactor of CRL3KEAP1 and CRL2VHL Ubiquitin Ligase Complexes Mediates Reciprocal Regulation of NRF2 and HIF-1α Proteins. Biochim. Biophys. Acta Mol. Cell Res..

[71] Zhang C., Peng Z., Zhu M., Wang P., Du X., Li X., Liu Y., Jin Y., McNutt M.A., Yin Y. (2016). USP9X Destabilizes PVHL and Promotes Cell Proliferation. Oncotarget.

[72] Kim E.B., Fang X., Fushan A.A., Huang Z., Lobanov A.V., Han L., Marino S.M., Sun X., Turanov A.A., Yang P. (2011). Genome Sequencing Reveals Insights into Physiology and Longevity of the Naked Mole Rat. Nature.

[73] Xiao B., Wang S., Yang G., Sun X., Zhao S., Lin L., Cheng J., Yang W., Cong W., Sun W. (2017). HIF-1α Contributes to Hypoxia Adaptation of the Naked Mole Rat. Oncotarget.

[74] Hawkins L.J., Hadj-Moussa H., Nguyen V.C., Pamenter M.E., Storey K.B. (2019). Naked Mole Rats Activate Neuroprotective Proteins during Hypoxia. J. Exp. Zool. A Ecol. Integr. Physiol..

[75] D’ignazio L., Rocha S. (2016). Hypoxia Induced NF-ΚB. Cells.

[76] Yang H., Minamishima Y.A., Yan Q., Schlisio S., Ebert B.L., Zhang X., Zhang L., Kim W.Y., Olumi A.F., Kaelin W.G. (2007). PVHL Acts as an Adaptor to Promote the Inhibitory Phosphorylation of the NF-ΚB Agonist Card9 by CK2. Mol. Cell.

[77] Courtois G. (2008). Tumor Suppressor CYLD: Negative Regulation of NF-ΚB Signaling and More. Cell. Mol. Life Sci..

[78] An J., Mo D., Liu H., Veena M.S., Srivatsan E.S., Massoumi R., Rettig M.B. (2008). Inactivation of the CYLD Deubiquitinase by HPV E6 Mediates Hypoxia-Induced NF-KappaB Activation. Cancer Cell.

[79] Bhardwaj A., Panepinto M.C., Ueberheide B., Neel B.G. (2025). A Mechanism for Hypoxia-Induced Inflammatory Cell Death in Cancer. Nature.

[80] Guo J., Shinriki S., Su Y., Nakamura T., Hayashi M., Tsuda Y., Murakami Y., Tasaki M., Hide T., Takezaki T. (2014). Hypoxia Suppresses Cylindromatosis (CYLD) Expression to Promote Inflammation in Glioblastoma: Possible Link to Acquired Resistance to Anti-VEGF Therapy. Oncotarget.

[81] Mathis B., Lai Y., Qu C., Janicki J., Cui T. (2015). CYLD-Mediated Signaling and Diseases. Curr. Drug Targets.

[82] Hadj-Moussa H., Chiasson S., Cheng H., Eaton L., Storey K.B., Pamenter M.E. (2021). MicroRNA-Mediated Inhibition of AMPK Coordinates Tissue-Specific Downregulation of Skeletal Muscle Metabolism in Hypoxic Naked Mole-Rats. J. Exp. Biol..

[83] Chen X., Li X., Zhang W., He J., Xu B., Lei B., Wang Z., Cates C., Rousselle T., Li J. (2018). Activation of AMPK Inhibits Inflammatory Response during Hypoxia and Reoxygenation through Modulating JNK-Mediated NF-ΚB Pathway. Metabolism.

[84] Kim S.J., Jung H.J., Lim C.J. (2013). Reactive Oxygen Species-Dependent down-Regulation of Tumor Suppressor Genes PTEN, USP28, DRAM, TIGAR, and CYLD under Oxidative Stress. Biochem. Genet..

[85] Kowalczyk A., Partha R., Clark N.L., Chikina M. (2020). Pan-Mammalian Analysis of Molecular Constraints Underlying Extended Lifespan. eLife.

[86] MacRae S.L., Croken M.M.K., Calder R.B., Aliper A., Milholland B., White R.R., Zhavoronkov A., Gladyshev V.N., Seluanov A., Gorbunova V. (2015). DNA Repair in Species with Extreme Lifespan Differences. Aging.

[87] Stead E.R., Bjedov I. (2021). Balancing DNA Repair to Prevent Ageing and Cancer. Exp. Cell Res..

[88] Gorbunova V., Seluanov A. (2016). DNA Double Strand Break Repair, Aging and the Chromatin Connection. Mutat. Res.-Fundam. Mol. Mech. Mutagen..

[89] Evdokimov A., Kutuzov M., Petruseva I., Lukjanchikova N., Kashina E., Kolova E., Zemerova T., Romanenko S., Perelman P., Prokopov D. (2018). Naked Mole Rat Cells Display More Efficient Excision Repair than Mouse Cells. Aging.

[90] Correale S., De Paola I., Morgillo C.M., Federico A., Zaccaro L., Pallante P., Galeone A., Fusco A., Pedone E., Luque F.J. (2014). Structural Model of the HUbA1-UbcH10 Quaternary Complex: In Silico and Experimental Analysis of the Protein-Protein Interactions between E1, E2 and Ubiquitin. PLoS ONE.

[91] Zhao Y., Long M.J.C., Wang Y., Zhang S., Aye Y. (2018). Ube2V2 Is a Rosetta Stone Bridging Redox and Ubiquitin Codes, Coordinating DNA Damage Responses. ACS Cent. Sci..

[92] Triplett J.C., Tramutola A., Swomley A., Kirk J., Grimes K., Lewis K., Orr M., Rodriguez K., Cai J., Klein J.B. (2015). Age-Related Changes in the Proteostasis Network in the Brain of the Naked Mole-Rat: Implications Promoting Healthy Longevity. Biochim. Biophys. Acta (BBA)-Mol. Basis Dis..

[93] Bekker-Jensen S., Mailand N. (2011). The Ubiquitin- and SUMO-Dependent Signaling Response to DNA Double-Strand Breaks. FEBS Lett..

[94] Tang J.B., Greenberg R.A. (2010). Connecting the Dots: Interplay between Ubiquitylation and SUMOylation at DNA Double-Strand Breaks. Genes Cancer.

[95] Gieni R.S., Ismail I.H., Campbell S., Hendzel M.J. (2011). Polycomb Group Proteins in the DNA Damage Response: A Link between Radiation Resistance and “Stemness”. Cell Cycle.

[96] Bekker-Jensen S., Danielsen J.R., Fugger K., Gromova I., Nerstedt A., Bartek J., Lukas J., Mailand N. (2010). HERC2 Coordinates Ubiquitin-Dependent Assembly of DNA Repair Factors on Damaged Chromosomes. Nat. Cell Biol..

[97] Danielsen J.R., Povlsen L.K., Villumsen B.H., Streicher W., Nilsson J., Wikström M., Bekker-Jensen S., Mailand N. (2012). DNA Damage-Inducible SUMOylation of HERC2 Promotes RNF8 Binding via a Novel SUMO-Binding Zinc Finger. J. Cell Biol..

[98] Zhang Z., Yang H., Wang H. (2014). The Histone H2A Deubiquitinase USP16 Interacts with HERC2 and Fine-Tunes Cellular Response to DNA Damage. J. Biol. Chem..

[99] Pinto É.S.M., Krause M.J., Dorn M., Feltes B.C. (2023). The Nucleotide Excision Repair Proteins through the Lens of Molecular Dynamics Simulations. DNA Repair.

[100] Sorokin A.V., Kim E.R., Ovchinnikov L.P. (2009). Proteasome System of Protein Degradation and Processing. Biochemistry.

[101] Petruseva I.O., Evdokimov A.N., Lavrik O.I. (2017). Genome Stability Maintenance in Naked Mole-Rat. Acta Naturae.

[102] Liguori I., Russo G., Curcio F., Bulli G., Aran L., Della-Morte D., Gargiulo G., Testa G., Cacciatore F., Bonaduce D. (2018). Oxidative Stress, Aging, and Diseases. Clin. Interv. Aging.

[103] Hajam Y.A., Rani R., Ganie S.Y., Sheikh T.A., Javaid D., Qadri S.S., Pramodh S., Alsulimani A., Alkhanani M.F., Harakeh S. (2022). Oxidative Stress in Human Pathology and Aging: Molecular Mechanisms and Perspectives. Cells.

[104] Ogiso Y., Tomida A., Kim H.D., Tsuruo T. (1999). Glucose Starvation and Hypoxia Induce Nuclear Accumulation of Proteasome in Cancer Cells. Biochem. Biophys. Res. Commun..

[105] Barrientos A. (2012). Complementary Roles of Mitochondrial Respiration and ROS Signaling on Cellular Aging and Longevity. Aging.

[106] Cedikova M., Pitule P., Kripnerova M., Markova M., Kuncová J. (2016). Multiple Roles of Mitochondria in Aging Processes. Physiol. Res..

[107] Kong Y., Trabucco S.E., Zhang H. (2014). Oxidative Stress, Mitochondrial Dysfunction and the Mitochondria Theory of Aging. Interdiscip. Top. Gerontol..

[108] Cadenas E., Davies K.J.A. (2000). Mitochondrial Free Radical Generation, Oxidative Stress, and Aging. Free Radic. Biol. Med..

[109] Sulkshane P., Ram J., Thakur A., Reis N., Kleifeld O., Glickman M.H. (2021). Ubiquitination and Receptor-Mediated Mitophagy Converge to Eliminate Oxidation-Damaged Mitochondria during Hypoxia. Redox Biol..

[110] Lee J., Giordano S., Zhang J. (2012). Autophagy, Mitochondria and Oxidative Stress: Cross-Talk and Redox Signalling. Biochem. J..

[111] Li A., Gao M., Liu B., Qin Y., Chen L., Liu H., Wu H., Gong G. (2022). Mitochondrial Autophagy: Molecular Mechanisms and Implications for Cardiovascular Disease. Cell Death Dis..

[112] Vays V., Vangely I., Eldarov C., Holtze S., Hildebrandt T., Bakeeva L., Skulachev V. (2021). Progressive Reorganization of Mitochondrial Apparatus in Aging Skeletal Muscle of Naked Mole Rats (*Heterocephalus glaber*) as Revealed by Electron Microscopy: Potential Role in Continual Maintenance of Muscle Activity. Aging.

[113] Popov N.A., Skulachev V.P. (2019). Neotenic Traits in *Heterocephalus glaber* and *Homo sapiens*. Biochemistry.

[114] Yap K.N., Wong H.S., Ramanathan C., Rodriguez-Wagner C.A., Roberts M.D., Freeman D.A., Buffenstein R., Zhang Y. (2022). Naked Mole-Rat and Damaraland Mole-Rat Exhibit Lower Respiration in Mitochondria, Cellular and Organismal Levels. Biochim. Biophys. Acta Bioenerg..

[115] Stoll E.A., Karapavlovic N., Rosa H., Woodmass M., Rygiel K., White K., Turnbull D.M., Faulkes C.G. (2016). Naked Mole-Rats Maintain Healthy Skeletal Muscle and Complex IV Mitochondrial Enzyme Function into Old Age. Aging.

[116] Bakeeva L., Vays V., Vangeli I., Eldarov C., Holtze S., Hildebrandt T., Skulachev V. (2019). Delayed Onset of Age-Dependent Changes in Ultrastructure of Myocardial Mitochondria as One of the Neotenic Features in Naked Mole Rats (*Heterocephalus glaber*). Int. J. Mol. Sci..

[117] Babbar M., Basu S., Yang B., Croteau D.L., Bohr V.A. (2020). Mitophagy and DNA Damage Signaling in Human Aging. Mech. Ageing Dev..

[118] Chaudhary P., Suryakumar G., Prasad R., Singh S.N., Ali S., Ilavazhagan G. (2012). Chronic Hypobaric Hypoxia Mediated Skeletal Muscle Atrophy: Role of Ubiquitin-Proteasome Pathway and Calpains. Mol. Cell Biochem..

[119] Debevec T., Ganse B., Mittag U., Eiken O., Mekjavic I.B., Rittweger J. (2018). Hypoxia Aggravates Inactivity-Related Muscle Wasting. Front. Physiol..

[120] Slot I.G.M., Schols A.M.W.J., de Theije C.C., Snepvangers F.J.M., Gosker H.R. (2016). Alterations in Skeletal Muscle Oxidative Phenotype in Mice Exposed to 3 Weeks of Normobaric Hypoxia. J. Cell Physiol..

[121] Lewis K.N., Mele J., Hayes J.D., Buffenstein R. (2010). Nrf2, a Guardian of Healthspan and Gatekeeper of Species Longevity. Integr. Comp. Biol..

[122] Lewis K.N., Wason E., Edrey Y.H., Kristan D.M., Nevo E., Buffenstein R. (2015). Regulation of Nrf2 Signaling and Longevity in Naturally Long-Lived Rodents. Proc. Natl. Acad. Sci. USA.

[123] Keane M., Craig T., Alföldi J., Berlin A.M., Johnson J., Seluanov A., Gorbunova V., Di Palma F., Lindblad-Toh K., Church G.M. (2014). The Naked Mole Rat Genome Resource: Facilitating Analyses of Cancer and Longevity-Related Adaptations. Bioinformatics.

[124] Chiu R.K., Brun J., Ramaekers C., Theys J., Weng L., Lambin P., Gray D.A., Wouters B.G. (2006). Lysine 63-Polyubiquitination Guards against Translesion Synthesis–Induced Mutations. PLoS Genet..

[125] Chang J., Knowlton A.A., Wasser J.S. (2000). Expression of Heat Shock Proteins in Turtle and Mammal Hearts: Relationship to Anoxia Tolerance. Am. J. Physiol. Regul. Integr. Comp. Physiol..

[126] Woods A.K., Storey K.B. (2005). Effects of Hibernation on Multicatalytic Proteinase Complex in Thirteen-Lined Ground Squirrels, *Spermophilus tridecemlineatus*. Mol. Cell Biochem..

[127] Hawkins L.J., Storey K.B. (2017). Improved High-Throughput Quantification of Luminescent Microplate Assays Using a Common Western-Blot Imaging System. MethodsX.

[128] Eaton S.L., Roche S.L., Llavero Hurtado M., Oldknow K.J., Farquharson C., Gillingwater T.H., Wishart T.M. (2013). Total Protein Analysis as a Reliable Loading Control for Quantitative Fluorescent Western Blotting. PLoS ONE.

[129] Zhang J., Storey K.B. (2016). RBioplot: An Easy-to-Use R Pipeline for Automated Statistical Analysis and Data Visualization in Molecular Biology and Biochemistry. PeerJ.

[130] Pena E., El Alam S., Siques P., Brito J. (2022). Oxidative Stress and Diseases Associated with High-Altitude Exposure. Antioxidants.

[131] Nathaniel T.I., Otukonyong E.E., Okon M., Chaves J., Cochran T., Nathaniel A.I. (2013). Metabolic Regulatory Clues from the Naked Mole Rat: Toward Brain Regulatory Functions during Stroke. Brain Res. Bull..

[132] Burtscher J., Mallet R.T., Pialoux V., Millet G.P., Burtscher M. (2022). Adaptive Responses to Hypoxia and/or Hyperoxia in Humans. Antioxid. Redox Signal..

[133] Li K., He C. (2019). Gastric Mucosal Lesions in Tibetans with High-Altitude Polycythemia Show Increased HIF-1A Expression and ROS Production. Biomed. Res. Int..

[134] Sharma S., Sandhir R., Ganju L., Kumar B., Singh Y. (2023). Unique Mutations in Mitochondrial DNA and Associated Pathways Involved in High Altitude Pulmonary Edema Susceptibility in Indian Lowlanders. J. Biomol. Struct. Dyn..

[135] Mallet R.T., Burtscher J., Pialoux V., Pasha Q., Ahmad Y., Millet G.P., Burtscher M. (2023). Molecular Mechanisms of High-Altitude Acclimatization. Int. J. Mol. Sci..

[136] Li H.S., Zhou Y.N., Li L., Li S.F., Long D., Chen X.L., Zhang J.B., Feng L., Li Y.P. (2019). HIF-1α Protects against Oxidative Stress by Directly Targeting Mitochondria. Redox Biol..

[137] Favier F.B., Britto F.A., Freyssenet D.G., Bigard X.A., Benoit H. (2015). HIF-1-Driven Skeletal Muscle Adaptations to Chronic Hypoxia: Molecular Insights into Muscle Physiology. Cell. Mol. Life Sci..

[138] Hoppeler H., Vogt M., Weibel E.R., Flück M. (2003). Response of Skeletal Muscle Mitochonrial to Hypoxia. Exp. Physiol..

[139] Murray A.J., Montgomery H.E., Feelisch M., Grocott M.P.W., Martin D.S. (2018). Metabolic Adjustment to High-Altitude Hypoxia: From Genetic Signals to Physiological Implications. Biochem. Soc. Trans..

