# Supplementary figures and images for: Hypoxia Increases Cardiac Proteasomal Activity and Differentially Modulates Cullin-RING E3 Ligases in the Naked Mole-Rat Heterocephalus glaber
Source: Muscles. 2026 Jan 14;5(1):6. doi: 10.3390/muscles5010006 (PMC12821419; doi:10.3390/muscles5010006)

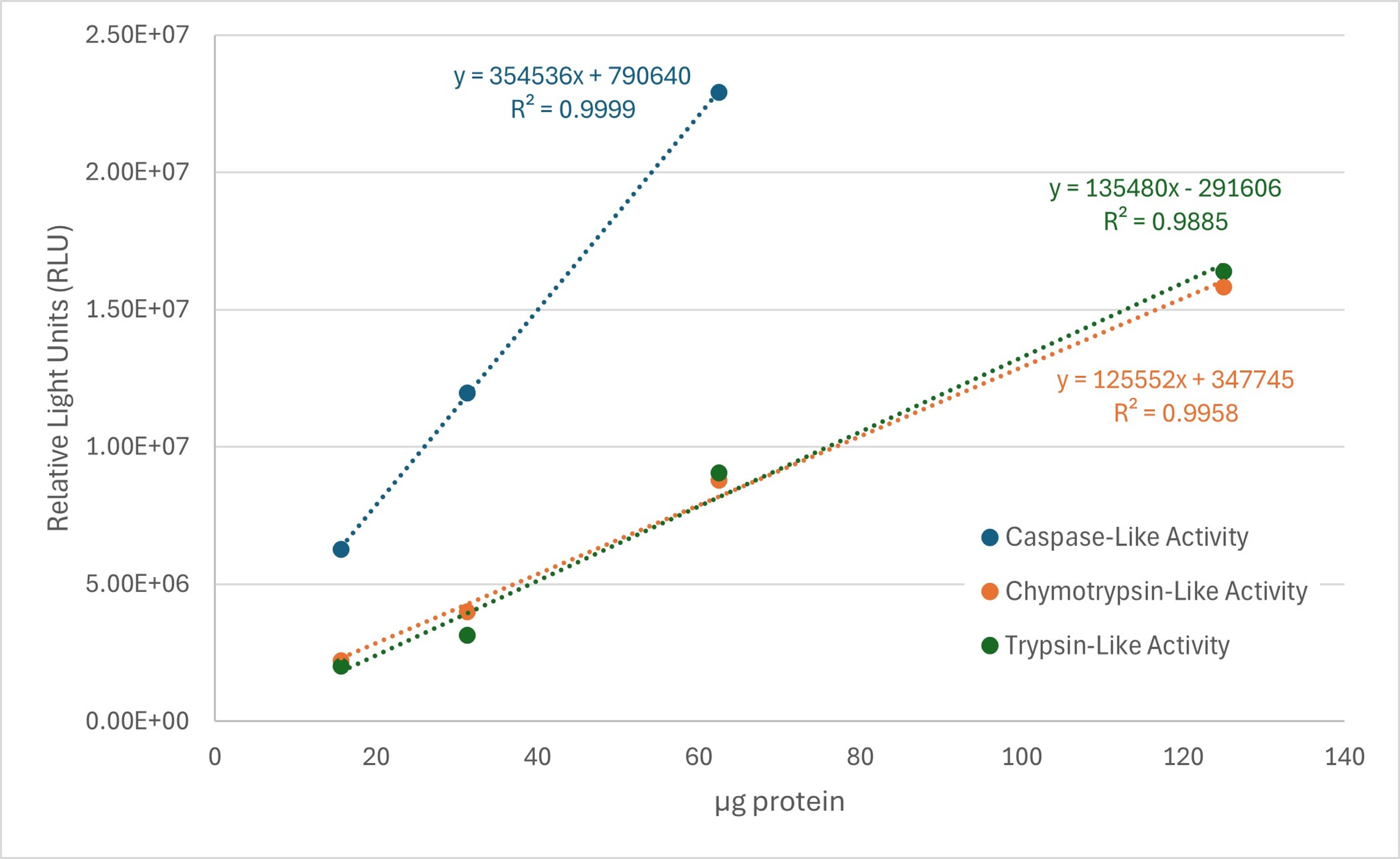

Supplement: Supplementary file 1 [file muscles-05-00006-s001.zip › Figure S1 - Standard curve for proteasomal assay.jpg]

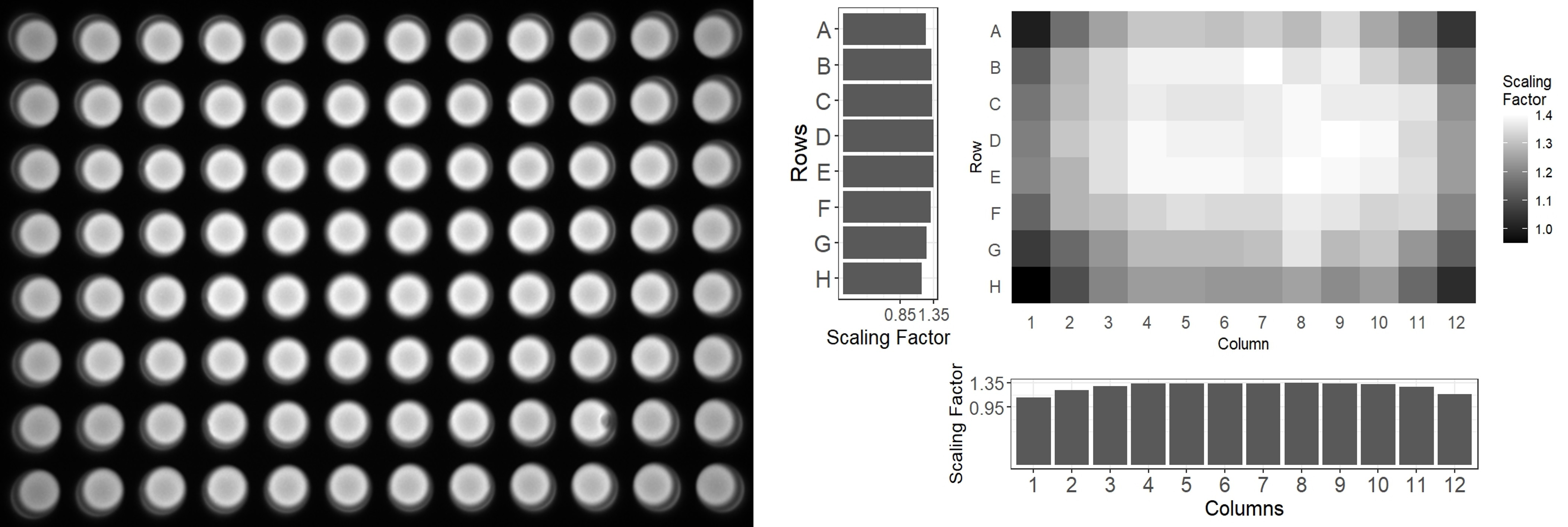

Supplement: Supplementary file 1 [file muscles-05-00006-s001.zip › Figure S2 - Well scaling factors for proteasomal assay.jpg]
